# Microvascular ALT-Flap Reconstruction for Distal Forearm and Hand Defects: Outcomes and Single-Case Application of a Bone-Anchored Venous Anastomosis

**DOI:** 10.3390/jcm14196807

**Published:** 2025-09-26

**Authors:** Adrian Matthias Vater, Matthias Michael Aitzetmüller-Klietz, Philipp Edmund Lamby, Julia Stanger, Rainer Meffert, Karsten Schmidt, Michael Georg Jakubietz, Rafael Gregor Jakubietz

**Affiliations:** 1 Department of Plastic, Aestethic, Hand and Reconstructive Surgery, Medizincampus Niederbayern, University of Regensburg, General Hospital Passau, Innstraße 76, 94032 Passau, Germany; 2 Department of Trauma-, Hand-, Plastic and Reconstructive Surgery, University Hospital Würzburg, Oberdürrbacher Straße 6, 97080 Würzburg, Germany; 3 Department of Trauma, Hand and Reconstructive Surgery, Division of Plastic Surgery, University Hospital Muenster, 48149 Muenster, Germany; 4 Division of Plastic Surgery, Institute of Musculoskeletal Medicine, University Hospital Muenster, 48149 Muenster, Germany

**Keywords:** anterior lateral thigh, free flap, upper extremity, venous congestion

## Abstract

**Background:** Reconstruction of distal forearm and hand soft tissue defects remains a complex surgical challenge due to the functional and aesthetic significance of the region. Several flap options have been established such as the posterior interosseous artery flap (PIA) or temporalis fascia flap (TFF), yet the anterolateral thigh flap (ALT) has gained increasing attention for its versatility and favorable risk profile. **Methods:** We retrospectively analyzed 12 patients (7 males, 5 females; mean age 51.8 years) who underwent free microvascular ALT reconstruction for distal forearm and hand defects between May 2020 and May 2025. Etiologies included infection, chemical burns, explosion injuries, and traffic accidents. The mean defect size was 75.4 cm^2^, and the average operative time was 217 min. Secondary flap thinning was performed in eight cases. In one patient without available recipient veins, a pedicle vein was anastomosed using a coupler device anchored into a cortical window of the distal radius to establish venous outflow via the bone marrow. **Results:** All flaps demonstrated complete survival with successful integration. Minor complications included transient venous congestion in one case and superficial wound dehiscence in four cases. Functional outcomes were favorable, with postoperative hand function rated as very good in 10 of 12 patients at follow-up. The bone-anchored venous anastomosis provided effective venous drainage in the salvage case. **Conclusions:** The free microvascular ALT is a reliable and highly adaptable method for distal forearm and hand reconstruction. It provides excellent soft tissue coverage, allows for secondary contouring, and achieves both functional and aesthetic goals. Furthermore, intraosseous venous anastomosis using a coupler device might represent a novel adjunct that may expand reconstructive options in cases with absent or unusable recipient veins.

## 1. Introduction

Soft tissue defects of the distal forearm and hand represent some of the most challenging problems in reconstructive surgery. These regions are essential for fine motor control, tactile sensation, and social interaction, yet are highly vulnerable to trauma, tumor resection, infection, and burns. Such injuries can cause severe functional impairment and aesthetic disfigurement. The anatomy of the distal forearm and hand, which is characterized by thin, mobile skin, limited soft tissue coverage, and the close proximity of tendons, joints, and bones, demands precise reconstructive planning. Successful reconstruction must achieve durability, pliability, and aesthetic integration while preserving or restoring function [1,2].

The primary goals of reconstruction are to restore form and re-establish hand and wrist function. Ideal coverage requires skin with a good color and texture match, sufficient mobility for joint motion, and minimal donor site morbidity to support overall recovery. Local and regional flaps, such as the posterior interosseous artery (PIA) flap or pedicled groin flap, remain valuable options for smaller defects. However, they are often inadequate for extensive or composite defects, which require greater tissue volume and adaptability. In such cases, free flaps have become the standard of care, with the temporalis fascia flap (TFF) and anterolateral thigh (ALT) flap representing two versatile choices [3,4,5,6,7].

Among these, the ALT has gained widespread recognition due to its large and customizable skin paddle, long vascular pedicle, and relatively low donor site morbidity. It provides reliable coverage, sufficient bulk that can be secondarily contoured, and durable long-term outcomes in both functional and aesthetic terms [8,9]. Consequently, the ALT has become a workhorse for reconstruction of distal forearm and hand defects.

A persistent challenge in microsurgical hand reconstruction, however, is the availability of suitable recipient veins. Venous insufficiency remains a leading cause of flap compromise, especially in cases of severe trauma or infection where recipient veins are absent or unusable [10]. To address this limitation, we introduce a novel technique in which a flap pedicle vein is anastomosed to a venous coupler device anchored directly into a K-wire-prepared cortical window of the distal radius. This approach enables venous outflow through the vascularized bone marrow, thereby improving backflow and reducing the risk of venous congestion. It expands the reconstructive armamentarium in situations where conventional venous anastomosis is not feasible.

The present study reports on 12 consecutive patients who underwent free ALT reconstruction for distal forearm and hand defects. In addition to evaluating flap survival, functional outcomes, and donor site morbidity, we highlight the feasibility and clinical significance of this novel bone-anchored coupler anastomosis as a salvage option for establishing reliable venous drainage in otherwise compromised cases.

## 2. Materials and Methods

### 2.1. Study Design

Between May 2020 and May 2025, 12 patients with complex soft tissue defects of the distal forearm and hand underwent free anterolateral thigh (ALT) flap reconstruction at the University Hospital Würzburg and the General Hospital Passau. This retrospective study included all cases with full-thickness soft tissue loss and exposure of tendons or bone.

Inclusion criteria were absence of suitable local flap options, adequate donor site integrity, and favorable vascular anatomy for microvascular anastomosis. Patients with systemic conditions contraindicating microsurgery, such as uncontrolled diabetes mellitus or severe peripheral vascular disease, were excluded.

### 2.2. Patient Collective

The cohort comprised 12 patients (7 men, 5 women) with a mean age of 51.8 years. Defects resulted from infection in 4 cases (2 insect bites, 1 cat bite, 1 unknown origin), chemical burns in 3 cases (1 from baker’s lye, 2 from cleaning agents), and explosion injuries in 3 cases (2 from gunshots, 1 from an air-conditioning explosion). Two additional patients presented with post-traumatic defects after road traffic accidents (1 bicycle, 1 car).

In one patient, a proximal row carpectomy (PRC) was performed preoperatively due to severe infection with septic osteomyelitis of the lunate and scaphoid. In another case, a gun explosion injury necessitated amputation of the first to third rays before soft tissue reconstruction. Notably, no fractures of the forearm or hand were observed in this cohort (Table 1).

### 2.3. Preoperative Assessment

All patients underwent comprehensive preoperative evaluation to confirm suitability for ALT reconstruction. Perforators of the lateral circumflex femoral artery were mapped using Doppler ultrasonography to guide flap design. CT angiography was performed to assess the vascular anatomy of potential recipient vessels, typically the radial or ulnar arteries. Allen’s test was conducted to confirm patency of both radial and ulnar arteries, ensuring adequate hand perfusion in case of unilateral arterial sacrifice.

### 2.4. Surgical Technique

**Figure 1:**
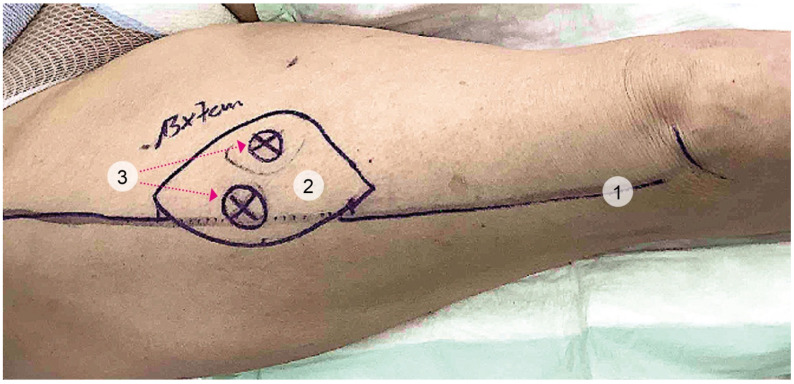
Preoperative markings. 1: line from superior anterior iliac spine to lateral margin of patella. 2: size-matched ALT. 3: Doppler-located perforators.

Flap design was based on preoperative perforator mapping. Dimensions were tailored to defect size, and in 7 of 12 patients fascia lata was included to provide additional volume (Figure 1). Flaps were elevated without primary thinning to preserve vascularity and transferred using microsurgical techniques. Arterial inflow was established by end-to-end anastomosis to either the radial or ulnar artery, depending on availability.

**Figure 2:**
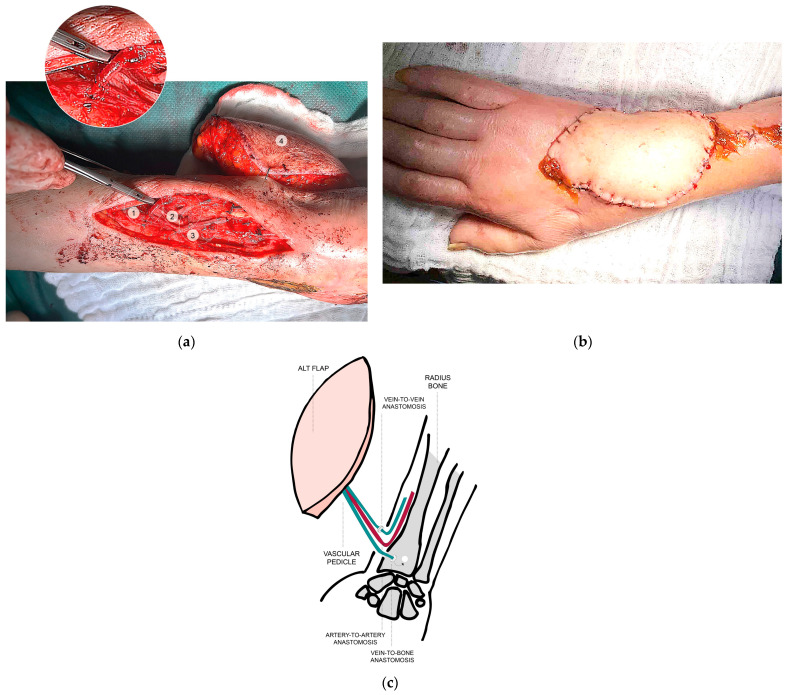
(**a**) Intraoperative findings with venous anastomosis into the radius bone. 1: vein-to-bone-coupler-anastomosis. 2: arterial anastomosis. 3: vein-to-vein-coupler-anastomosis. 4: ALT. (**b**) Initial postoperative result. (**c**) Concept of vein-to-bone coupler anastomosis.

In one case, where no suitable venous recipient vessels were available, we applied a novel strategy: one pedicle vein was anastomosed with a subcutaneous vein; by persistent venous congestion of the ALT, the other was anastomosed with a coupler device inserted into a K-wire-prepared, size-matched cortical window on the dorsum of the radius. Further fixation with bone wax and fibrin glue further stabilized anastomosis and this fixation secured the coupler ring within the bone, enabling sufficient venous drainage through the vascularized bone marrow and thereby augmenting venous backflow (Figure 2a–c).

After transfer, flap positioning was adjusted for optimal coverage and aesthetic contour. Secondary flap thinning was carried out in 8 patients, usually 8–12 weeks postoperatively, to refine contour and improve joint mobility. No primary thinning was performed at elevation to avoid compromising perfusion.

### 2.5. Postoperative Care

Postoperative management focused on flap monitoring and early rehabilitation. Flap perfusion was regularly assessed with Doppler ultrasonography, and patients were closely observed for signs of venous congestion, ischemia, or infection. Early mobilization was encouraged once flap stability was confirmed, typically starting 7 days postoperatively, to minimize stiffness and optimize functional recovery.

Wounds were monitored for infection or delayed healing, and complications were managed conservatively. Functional outcomes were evaluated at 6 months using clinical examination and patient-reported outcomes, including range of motion, grip strength, return-to-work time, sensory recovery, and overall satisfaction.

## 3. Results

### 3.1. Flap Survival and Complications

All 12 ALT survived without partial or total loss. No patient required emergent re-exploration for vascular compromise. Minor complications occurred in 5 patients: transient venous congestion in 1 case and superficial wound dehiscence in 4 cases. All were managed conservatively with dressings and local wound care, and no patient required secondary revision surgery. Secondary flap thinning was performed in 8 patients to optimize contour and joint mobility, while the remaining 4 achieved satisfactory results without revision (Table 2).

### 3.2. Functional Outcomes

At a mean follow-up of 12 months, functional outcomes were favorable in most cases. Hand function was rated as very good in 10 of 12 patients, with full or near-complete range of motion and excellent skin pliability. Two patients showed moderate residual stiffness, attributable to pre-existing joint damage from infection or trauma. Grip strength was comparable to the contralateral hand in 9 patients, while 3 experienced mild weakness that improved with time. The median return-to-work time was 42 days. Aesthetic outcomes were also positive, with most patients expressing high satisfaction with the appearance of the reconstructed hand and forearm (Table 2).

### 3.3. Donor Site Morbidity

Donor site morbidity was minimal. All donor sites were closed primarily without the need for skin grafting or secondary procedures. No patient developed significant functional impairment or visible deformity at the donor site, and postoperative discomfort was reported as minimal.

### 3.4. Sensory Recovery

Sensory recovery was limited in the absence of nerve coaptation. Protective sensation was achieved in four patients, while discriminative two-point sensation was documented in only one patient; the remaining flaps remained insensate. These findings are consistent with reports indicating that non-innervated free flaps generally provide only limited sensory return [10]. While protective sensation may be sufficient for basic hand functions, fine discriminative sensation is essential for delicate tasks. Recent studies, particularly in foot and ankle reconstruction, have shown that sensate ALT, when harvested with dedicated nerve branches, can restore both protective and two-point discrimination effectively [11]. Anatomical mapping of the ALT confirms the presence of reliable sensory nerve branches that can facilitate intentional innervation [12]. Future investigations should therefore explore routine nerve coaptation in ALT reconstructions of the hand, especially in patients with high functional demands (Table 2).

## 4. Discussion

Reconstructive surgery of the distal forearm and hand remains one of the most demanding areas within plastic and reconstructive surgery. The hand is not only functionally indispensable but also socially significant, as it plays a major role in non-verbal communication, personal identity, and quality of life. Successful reconstruction in this anatomical region requires a balance between providing durable soft tissue coverage and maintaining mobility, sensation, and cosmetic appearance. Over the past decades, various reconstructive strategies have been developed, ranging from local and regional flaps to free tissue transfer. Among these, the free anterolateral thigh (ALT) flap has become increasingly recognized as a reliable and versatile option.

### 4.1. Versatility and Coverage Options

The ALT is distinguished by its versatility. It can be harvested as a fasciocutaneous flap, musculocutaneous flap, or perforator flap, allowing the surgeon to tailor the design to the requirements of the defect. This adaptability provides an advantage over local flaps, which are frequently limited by size, arc of rotation, or tissue characteristics. For example, the posterior interosseous artery (PIA) flap can be useful in small-to-moderate dorsal hand defects but is restricted in its dimensions due to vascular constraints [13]. Similarly, pedicled groin flaps, while reliable, require prolonged immobilization and frequently lead to functional restrictions during the postoperative period. By contrast, the ALT can provide extensive coverage, and its pliable skin paddle can be modified to fit complex three-dimensional defects.

Previous studies have consistently demonstrated the ALT’s capacity for covering both small and extensive soft tissue defects of the upper extremity [14]. Its large and customizable skin paddle, combined with the option of including fascia lata or part of the vastus lateralis muscle when volume is required, provides unparalleled flexibility. In the current series, defects ranged from 40 to over 100 cm^2^, and the ALT could be tailored accordingly without compromising donor site closure. This flexibility underlines why the ALT has become a true “workhorse” in upper extremity reconstruction.

### 4.2. Donor Site Morbidity

Donor site morbidity is a decisive factor when selecting a flap, as functional and aesthetic impairment at the harvest site can undermine the benefits of the reconstruction. The thigh provides a relatively inconspicuous donor site, and in most cases primary closure is possible without grafting. This contrasts with radial forearm free flaps, where donor site morbidity including tendon exposure, need for skin grafting, and reduced wrist strength is well documented.

Compared with the temporalis fascia flap (TFF), which carries the risk of alopecia or visible scarring at the donor site [15,16], the ALT avoids cosmetically sensitive regions. Moreover, the functional sequelae are minimal, since harvesting from the thigh rarely compromises ambulation. In our series, all donor sites were closed primarily without additional procedures, and no patient reported long-term morbidity. This corroborates findings from other studies reporting low complication rates and high patient satisfaction following ALT harvest [15,16].

### 4.3. Reconstructive Reliability

Reliability is another hallmark of the ALT. The vascular anatomy of the descending branch of the lateral circumflex femoral artery is well studied and consistent, with multiple perforators suitable for flap harvest. The vascular pedicle is usually of sufficient length and caliber to allow tension-free microvascular anastomosis, even in distal locations such as the hand.

Our series confirmed the high survival rate of the ALT, with no partial or total losses. These results mirror findings from larger series and systematic reviews, where survival rates approach 95–100% [17]. By contrast, flaps such as the PIA are more prone to venous congestion and ischemia, particularly when harvested in larger dimensions. Furthermore, the anatomical variations in the vascular anatomy of the PIA may complicate its dissection and lead to donor-site morbidity [18].

### 4.4. Aesthetic and Functional Outcomes

Beyond survival, the quality of functional and aesthetic outcomes is decisive. The ALT provides skin that is relatively thin, pliable, and amenable to secondary thinning. This allows for contour refinement and a closer match to the texture and color of the hand and forearm. In our study, secondary flap thinning was performed in two-thirds of patients, yielding excellent contour and mobility.

**Figure 3:**
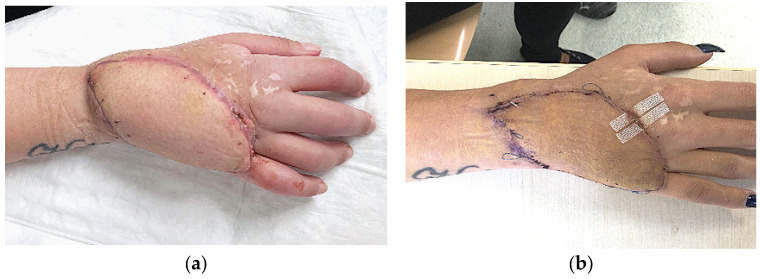
Early postoperative (**a**) and late postoperative (**b**) result after chemical burn injury.

**Figure 4:**
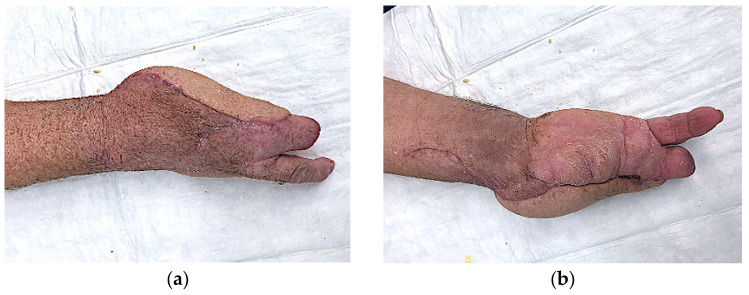
Postoperative dorsal (**a**) and volar (**b**) result after gunshot injury.

Functionally, most patients achieved full or near-full range of motion, with hand function rated as very good in 10 of 12 cases. Two patients demonstrated moderate functional limitations, but both were attributable to pre-existing conditions. In one, proximal row carpectomy (PRC) was required due to septic osteomyelitis, compromising wrist stability. In another, traumatic amputation of the first three rays precluded recovery of fine motor grip despite reliable coverage (Figure 3 and Figure 4). These examples highlight that flap survival alone does not determine outcome; preoperative pathology and anatomical loss significantly shape functional recovery.

Nevertheless, one important limitation of this study is that functional outcomes were not assessed with validated patient-reported outcome measures (PROMs) such as the Disabilities of the Arm, Shoulder and Hand (DASH) score or the Michigan Hand Questionnaire (MHQ)**.** While clinical evaluation and patient satisfaction were favorable, validated PROMs would provide a more standardized and comparable assessment of upper extremity function, quality of life, and disability. Future studies should therefore include such validated measures to strengthen the evaluation of postoperative outcomes.

Aesthetically, the ALT allowed satisfactory restoration in all patients. The skin paddle provided good texture and pliability, and patients reported high satisfaction with the appearance of their reconstructed hand. Compared with bulkier muscle flaps or less pliable fasciocutaneous flaps, the ALT offers superior contouring potential (Figure 3 and Figure 4) [14].

In terms of sensory recovery, our series confirmed that non-innervated ALTs provide only limited return, with most patients remaining insensate. This is consistent with earlier reports on upper limb reconstruction, which have demonstrated that sensate ALTs with nerve coaptation to the superficial radial nerve or other recipient branches can improve outcomes by restoring protective sensation and, in some cases, discriminative two-point perception [12]. A recent systematic review of upper limb free flaps further emphasized that sensory nerve coaptation is associated with improved tactile recovery, greater patient satisfaction, and enhanced functional integration, although outcomes remain variable [19]. Thus, while reliable for coverage, non-sensate flaps may fall short of optimal function in patients with high functional demands, underscoring the importance of considering nerve coaptation when planning ALT transfers to the hand.

More broadly, long-term studies of upper limb free flap reconstruction show that while flap survival exceeds 95%, functional recovery often lags behind structural success [18,19,20]. Patient-reported outcomes typically reveal residual deficits in fine motor skills, grip strength, and sensory function, even when coverage is stable and aesthetically acceptable. These findings highlight the need for integrating functional PROMs, sensory reinnervation and structured rehabilitation into future protocols for ALT reconstructions of the hand.

### 4.5. Complication Profile and Literature Context

Although free flap reconstruction is considered safe, it carries inherent risks. Our series reported only minor complications: transient venous congestion in one patient and wound dehiscence in four, all managed conservatively. Importantly, no re-explorations or flap losses occurred.

However, when situating our findings in the broader literature, it becomes clear that free flaps in the upper extremity carry somewhat higher perioperative complication rates compared with other regions. The meta-analysis by Zhang et al. (2019) [18] analyzing 283 flaps across 23 studies reported complication rates of 7% for infection, 7% for wound dehiscence, 6% for hematoma, 6% for seroma, and 6% for dysesthesia. These findings emphasize that while survival is excellent, vigilance regarding postoperative complications is critical, particularly in contaminated or traumatized wounds [18]. Lucattelli et al. (2021) further highlighted the high success of free flaps after sarcoma resections but likewise pointed to the need for standardized reporting of complications and functional outcomes [20].

### 4.6. Minimized Risk of Complications

While all surgical procedures carry inherent risks, the ALT demonstrates a relatively low complication profile compared to other flaps. Its predictable vascular anatomy and straightforward harvest make it less technically demanding than many alternatives. Nevertheless, venous outflow remains the most critical factor in preventing flap compromise [21].

### 4.7. Bone-Anchored Venous Anastomosis: Rationale and Clinical Implications

Reliable venous outflow is a key determinant of free flap survival, yet it often poses the greatest challenge in complex reconstructions. Venous congestion is a major cause of flap compromise, particularly in patients with severe trauma, infection, or multiple prior operations, where suitable recipient veins may be absent or unusable. Conventional salvage strategies such as interpositional vein grafts, end-to-side venous anastomoses, or arteriovenous loops are technically demanding, lengthen operative time, and are not always feasible in compromised recipient beds.

In this context, we employed a novel approach: anchoring a venous coupler into a K-wire-prepared cortical window of the distal radius to establish intraosseous venous outflow. The bone marrow cavity contains a rich sinusoidal venous network that communicates with systemic venous circulation, effectively functioning as a low-pressure venous reservoir. By connecting a flap vein directly to this vascularized cavity, we created an alternative drainage pathway when conventional options were unavailable.

This technique offers several theoretical advantages. The rigid fixation of the coupler within the cortical window minimizes kinking or torsion, common causes of venous thrombosis in soft tissue. The intraosseous space provides a broad, compliant venous bed, potentially reducing the risk of congestion. Furthermore, the procedure avoids prolonged vein grafting and is technically straightforward once the cortical window has been prepared and size-matched to the coupler.

Nevertheless, some limitations must be considered. Long-term patency of bone-anchored venous anastomoses remains unknown, and the possibility of intraosseous thrombosis has not yet been systematically studied. Local infection, particularly in osteomyelitic or contaminated wounds, represents another concern, although no septic complications occurred in our cohort. Technical precision is essential to ensure stable fixation and optimal flow.

To our knowledge, this represents one of the first clinical applications of bone-anchored venous outflow in free flap surgery. The successful outcome in our case suggests that intraosseous drainage can be a valuable adjunct in complex reconstructions with limited venous options. Future studies should investigate the hemodynamic characteristics of intraosseous venous outflow, histological integration at the bone–coupler interface, and long-term clinical outcomes. If validated, this technique may provide a reproducible and standardized salvage option in microsurgery, especially in upper extremity reconstruction where venous drainage remains a persistent challenge.

### 4.8. Future Directions

Improved microsurgical techniques remain a promising research avenue. Studies show that performing two venous anastomoses lowers thrombosis risk compared with a single anastomosis [20]. Our bone-anchored coupler method offers a novel alternative when dual venous outflow is not achievable. Further studies should clarify its indications and potential for broader application.

In addition, perfusion-optimized flap strategies using advanced imaging such as indocyanine green (ICG) angiography or 3D Doppler can provide real-time perfusion assessment and improve intraoperative decision-making [22]. Long-term multicenter studies focusing on function, sensation, and quality of life are also needed to better define the true impact of ALT reconstruction in the hand and forearm [23].

Postoperative rehabilitation is another underexplored area. Early mobilization, structured physiotherapy, and scar management are essential to prevent stiffness and maximize mobility. In our series, early mobilization was initiated approximately one week after surgery, which facilitated good recovery in most patients.

In cases with severe tissue loss or ray amputations, integration of hand prostheses into rehabilitation may provide additional benefits. Early prosthetic fitting can support grip, assist in daily activities, and reduce psychosocial distress. Coordinated care involving reconstructive surgeons, physiotherapists, and prosthetic specialists ensures optimal adaptation and function. While literature on prosthetic use after free flap reconstruction remains limited, our findings underscore the potential synergy between biological reconstruction and assistive technology.

## 5. Conclusions

The anterolateral thigh flap is a versatile, reliable, and aesthetically favorable option for reconstruction of complex distal forearm and hand defects. Compared with alternatives such as the PIA, pedicled groin flap, or TFF, it offers distinct advantages in versatility, donor site safety, aesthetic outcomes, and reconstructive reliability.

Our series confirms its promising survival and functional results, with minimal donor site morbidity. Furthermore, we introduce a novel adjunct: bone-anchored venous anastomosis using a coupler device fixed into the distal radius. This technique provides an additional outflow pathway via the vascularized bone marrow, offering a salvage option when conventional venous anastomosis is not feasible.

While larger studies are needed to validate its reproducibility and long-term outcomes, our findings suggest that this approach may expand the reconstructive armamentarium for complex upper extremity defects. Ultimately, the ALT, especially when combined with innovative microsurgical techniques and structured rehabilitation, remains an effective strategy for restoring both function and form in the distal forearm and hand.

## Figures and Tables

**Table 1:** Baseline data.

Total Number of Patients	12
Male	7
Female	5
Mean age	51.8 years
Infection (n)	4 (2 insect bites, 1 cat bite, 1 unknown)
Chemical burns (n)	3 (1 baker’s lye, 2 cleaning agents)
Explosion injuries (n)	3 (2 gunshots, 1 air-conditioning explosion)
Road traffic accidents (n)	2 (1 bicycle, 1 car accident)
Mean defect size	75.4 cm^2^
Average operative time	217 min
Follow-up range (months)	6–60 (mean 24)

**Table 2:** Results.

Category	FINDINGS
Flap survival	100% (12/12)
Re-exploration required	0% (0/12)
Minor complications	42% (5/12)
- Venous congestion	8% (1/12)
- Wound dehiscence	33% (4/12)
Secondary flap thinning	67% (8/12)
Good/very good hand function	83% (10/12)
Residual stiffness	17% (2/12)
Grip strength ≈ contralateral hand	75% (9/12)
Return-to-work (median)	42 days
Protective sensation	33% (4/12)
Discriminative sensation	8% (1/12)
Insensate flaps	58% (7/12)
Donor site morbidity	0% (0/12)

## Data Availability

All patient data were used anonymized and received by the patient chart in consent to the local ethical committee.

## References

[1] Chim H., Ng Z.Y., Carlsen B.T., Mohan A.T., Saint-Cyr M. (2014). Soft tissue coverage of the upper extremity: An overview. Hand Clin..

[2] Moshammer H., Schwarzl F., Haas F., Pierer G., Scharnagl E. (1998). Der Interossea posterior-Lappen--Ubersichtsarbeit und eigene Erfahrungen [The posterior interosseous flap--review and personal experience]. Handchir. Mikrochir. Plast. Chir..

[3] Lee G., Kim B., Jeon N., Yoon J., Hong K.Y., Lim S., Eo S. (2023). Versatility of the Posterior Interosseous Artery Flap: Emphasis on Powering Up the Toe Transfer. Hand.

[4] Amadei F., Fozzato S., Prevot L.B., Ciccarelli A., Bruno M., Basile G. (2023). The posterior interosseus artery flap (piaf) in reconstructive surgery of the hand: Strategies of use and medico-legal implications. Clin. Ter..

[5] Goertz O., Kapalschinski N., Daigeler A., Hirsch T., Homann H.H., Steinstraesser L., Lehnhardt M., Steinau H.U. (2012). The effectiveness of pedicled groin flaps in the treatment of hand defects: Results of 49 patients. J. Hand Surg. Am..

[6] Turan A. (2023). The Pedicled Sensate Osteocutaneous Groin Flap for Reconstruction of the Forearm and Hand. Ann. Plast. Surg..

[7] Upton J., Rogers C., Durham-Smith G., Swartz W.M. (1986). Clinical applications of free temporoparietal flaps in hand reconstruction. J. Hand Surg. Am..

[8] Adani R., Tarallo L., Marcoccio I., Cipriani R., Gelati C., Innocenti M. (2005). Hand reconstruction using the thin anterolateral thigh flap. Plast. Reconstr. Surg..

[9] Cheng L., Dai Q., Du W.L., Che K.X., Cao T.Y., Shen Y.M. (2025). Clinical Efficacy of Double Skin Paddle Anterolateral Thigh Flap in High-Voltage Electrical Burns of the Wrist and Hand. Ann. Plast. Surg..

[10] Sinis N., Lamia A., Gudrun H., Schoeller T., Werdin F. (2012). Sensory reinnervation of free flaps in reconstruction of the breast and the upper and lower extremities. Neural Regen. Res..

[11] Pan Z.H., Zhao Y.X., Ye X.H., Wang J.B., Li X.B. (2024). Surgical refinements and sensory and functional outcomes of using thinned sensate anterolateral thigh perforator flaps for foot and ankle reconstruction: A retrospective study. Medicine.

[12] Luenam S., Prugsawan K., Kosiyatrakul A., Chotanaphuti T., Sriya P. (2015). Neural Anatomy of the Anterolateral Thigh Flap. J. Hand Microsurg..

[13] Ren J., Lu L., Gao F., Liu B. (2021). The use of the posterior interosseous artery flap and anterolateral thigh flap for post-traumatic soft tissue reconstruction of the hand. Medicine.

[14] Adani R., Gaini S., Stelitano C. (2020). Free anterolateral thigh flap in hand and upper extremity reconstruction: A retrospective study. J. Hand Surg. Am..

[15] Wei F.-C., Mardini S. (2016). Free tissue transfer in the hand: Review of 33 years ofexperience. Hand Clin..

[16] Müller-Seubert W., Horch R.E., Schmidt V.F., Ludolph I., Schmitz M., Arkudas A. (2021). Retrospective analysis of free temporoparietal fascial flap for defect reconstruction of the hand and the distal upper extremity. Arch. Orthop. Trauma Surg..

[17] Zhou J., Zhang Y., He M., Zhang Z., Chen C., Zhang F. (2018). Comparing the posterior interosseous artery flap and anterolateral thigh flap for hand reconstruction: A systematic review and meta-analysis. Plast. Reconstr. Surg..

[18] Zhang Y., Gazyakan E., Bigdeli A.K., Will-Marks P., Kneser U., Hirche C. (2019). Soft tissue free flap for reconstruction of upper extremities: A meta-analysis on outcome and safety. Microsurgery.

[19] van Bekkum S., de Jong T., Zuidam M., Mureau M.A.M. (2020). Long-Term Quality of Life after Free Flap Upper Extremity Reconstruction for Traumatic Injuries. J. Reconstr. Microsurg..

[20] Lucattelli E., Lusetti I.L., Cipriani F., Innocenti A., De Santis G., Innocenti M. (2021). Reconstruction of upper limb soft-tissue defects after sarcoma resection with free flaps: A systematic review. J. Plast. Reconstr. Aesthetic Surg..

[21] Iamaguchi R., Burgos F., Silva G., Cho A., Nakamoto H., Takemura R., Wei T., de Rezende M., Mattar R. (2019). Do two venous anastomoses decrease venous thrombosis during limb reconstruction?. Clin. Hemorheol. Microcirc..

[22] Prantl L., Pfister K., Kubale R., Schmitt S., Stockhammer V., Jung W., Zorger N., Herold T., Nerlich M., Stehr A. (2007). Value of high resolution ultrasound and contrast enhanced US pulse inversion imaging for the evaluation of the vascular integrity of free-flap grafts. Clin. Hemorheol. Microcirc..

[23] Hagiga A., Aly M., Kadhum M., Christopoulos G. (2022). Functional and Aesthetic Outcomes of the Anterolateral Thigh Flap in Reconstruction of Upper Limb Defects: A Systematic Review. World J. Plast. Surg..

